# Can Three Screws and a Fibula be a Viable Treatment for Managing Neglected Femoral Neck Fracture in Trans-Femoral Amputees? – A Report of Two Cases

**DOI:** 10.7759/cureus.5682

**Published:** 2019-09-17

**Authors:** Prateek Behera, Lokesh SN, Ankit Khurana, Umesh Kumar Meena, Nirmal Raj Gopinathan

**Affiliations:** 1 Orthopaedics, All India Institute of Medical Sciences, Bhopal, IND; 2 Orthopaedics, Employess State Insurance Corporation Hospital, Chennai, IND; 3 Orthopaedics, All India Institute of Medical Sciences, Delhi, IND; 4 Orthopaedics, Sawai Man Singh Medical College, Jaipur, IND; 5 Orthopaedics, Post Graduate Institute of Medical Education and Research, Chandigarh, IND

**Keywords:** femoral neck non-union, cannulated screws, trans-femoral amputee, fibula autograft, free fibula, neglected injuries

## Abstract

Management of neglected femoral neck fracture in a trans-femoral amputee is difficult and challenging. There are limited options available for management of such a fracture. While arthroplasty (hemi or total) can be offered in older individuals, young patients should be offered an attempt of salvage of their native hips. Neglected femoral neck fracture in two young male patients who were trans-femoral amputees was managed by fixation through a Watson-Jones approach. Strategically placed Schanz screws and K-wires were used as joysticks for obtaining reduction and three 6.5mm cannulated screws were placed in a triangular fashion. An augmentation of the fixation was done with free fibula autograft placed in the center of the triangle. Union was achieved in both the cases. Patients were pain-free at the latest follow-up visit. Meticulous clinical and radiological evaluation is mandatory in multiply injured patients to avoid missing fractures. Fixation of neglected femoral neck fractures in young transfemoral amputees with three screws and a fibula can be considered a viable alternative to valgus osteotomy in cases where the stump is small for successful placement of the implant and where implant availability is an issue or the surgeon is comfortable in using screws and fibula for non-unions of femoral neck.

## Introduction

Femoral neck fractures may be seen alongside femoral shaft fractures, with a reported incidence of around 1-9% [1]. These fractures may be missed if appropriate clinical and radiological examinations are not done at the time of the patient’s presentation. This is especially true if there are any ‘distracting injuries’ in the same limb [2]. Presence of vascular injury in a patient may draw the attention of the physician towards them and an associated femoral neck fracture may actually be missed only to be diagnosed later.

Neglected femoral neck fracture in a trans-femoral amputee may be a new fracture in someone who is already an amputee, or rarely, it may be diagnosed in a patient who may have needed an amputation for some other reason and the femoral neck fracture might have been missed or neglected. While few case reports are available describing management of acute femoral neck fracture in trans-femoral (above knee) amputees, only one case report in English language describing management of neglected femoral neck fracture in above knee amputee could be retrieved [3-7]. Though there are various techniques of managing neglected femoral neck fractures with viable head in young adults, they are often managed satisfactorily by using non-vascularized fibula in combination with cannulated screws at many centers around the world.

Faced with the challenge of managing neglected femoral neck fractures in two young trans-femoral amputees, we fixed the fractures by using three partially threaded cancellous screws (PTCS) and a non-vascularized fibula auto-graft. Although other methods of treatment can also potentially be applied in similar situations we believe that in scenarios where proper implants are not available or the surgeon is familiar with the use of fibula auto-graft, this method can be a viable and useful alternative. The short-term results were satisfactory and this simple technique can be a useful tool in the armamentarium of orthopedic surgeons in management of similar fractures.

## Case presentation

**Figure 1:**
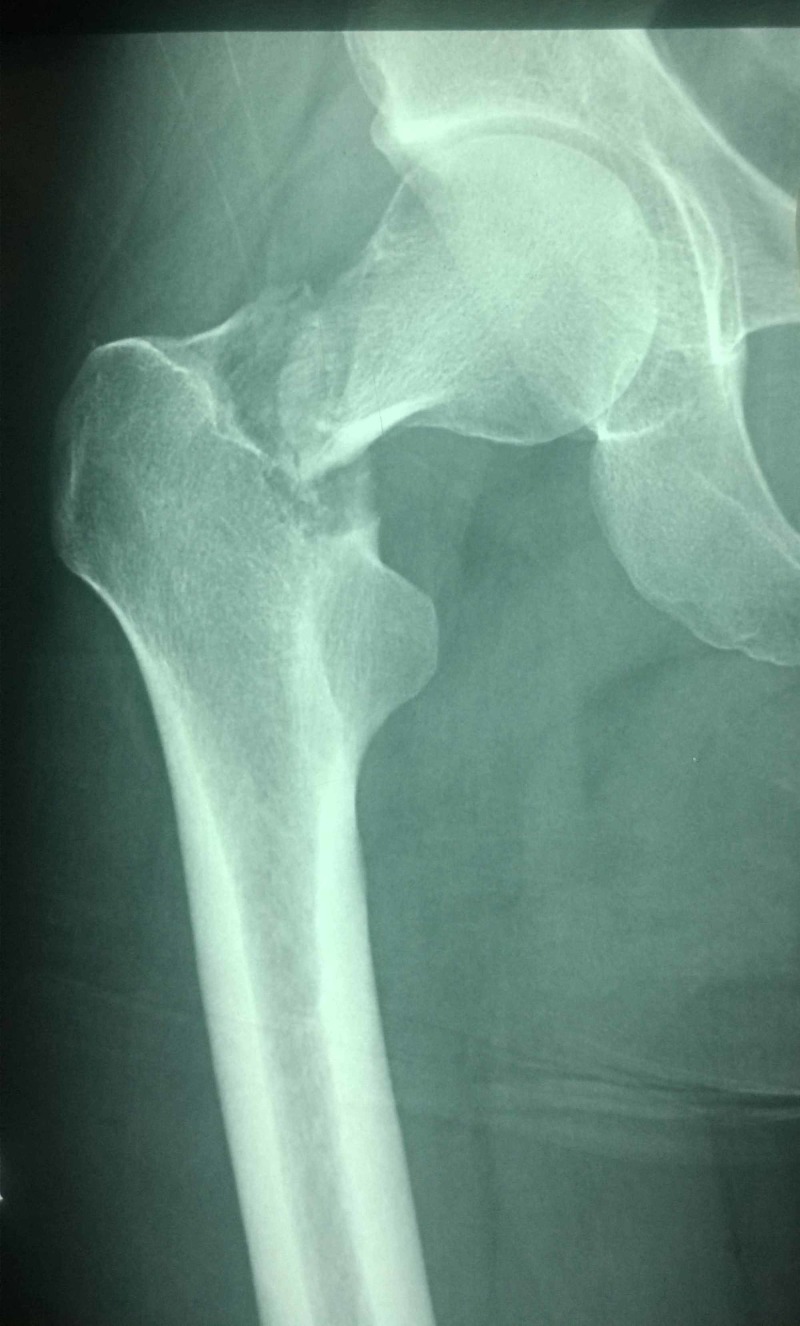
Antero-posterior radiograph of patient A showing right sided femoral neck fracture. The head appears to be rotated.

**Figure 2:**
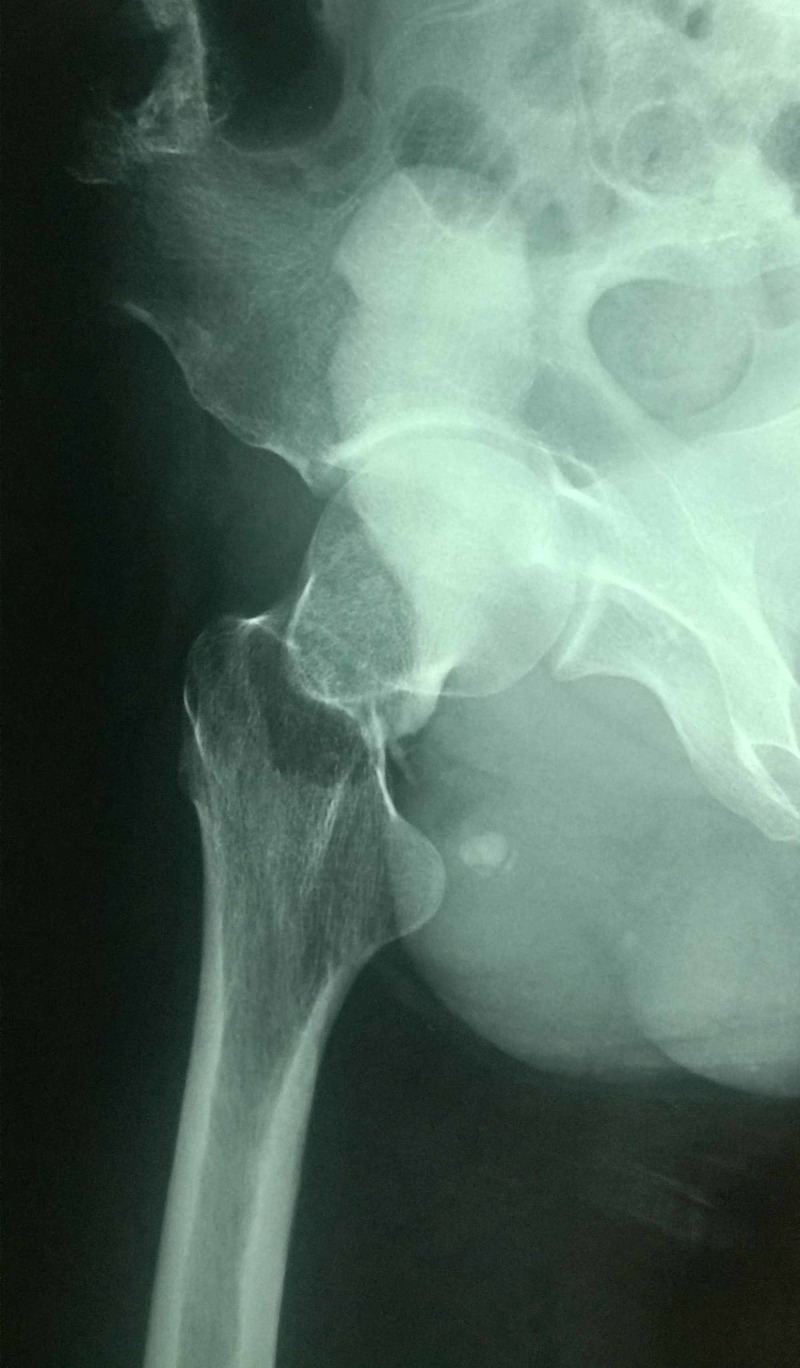
Lateral radiograph of patient A with the femoral neck fracture evident on it.

**Figure 3:**
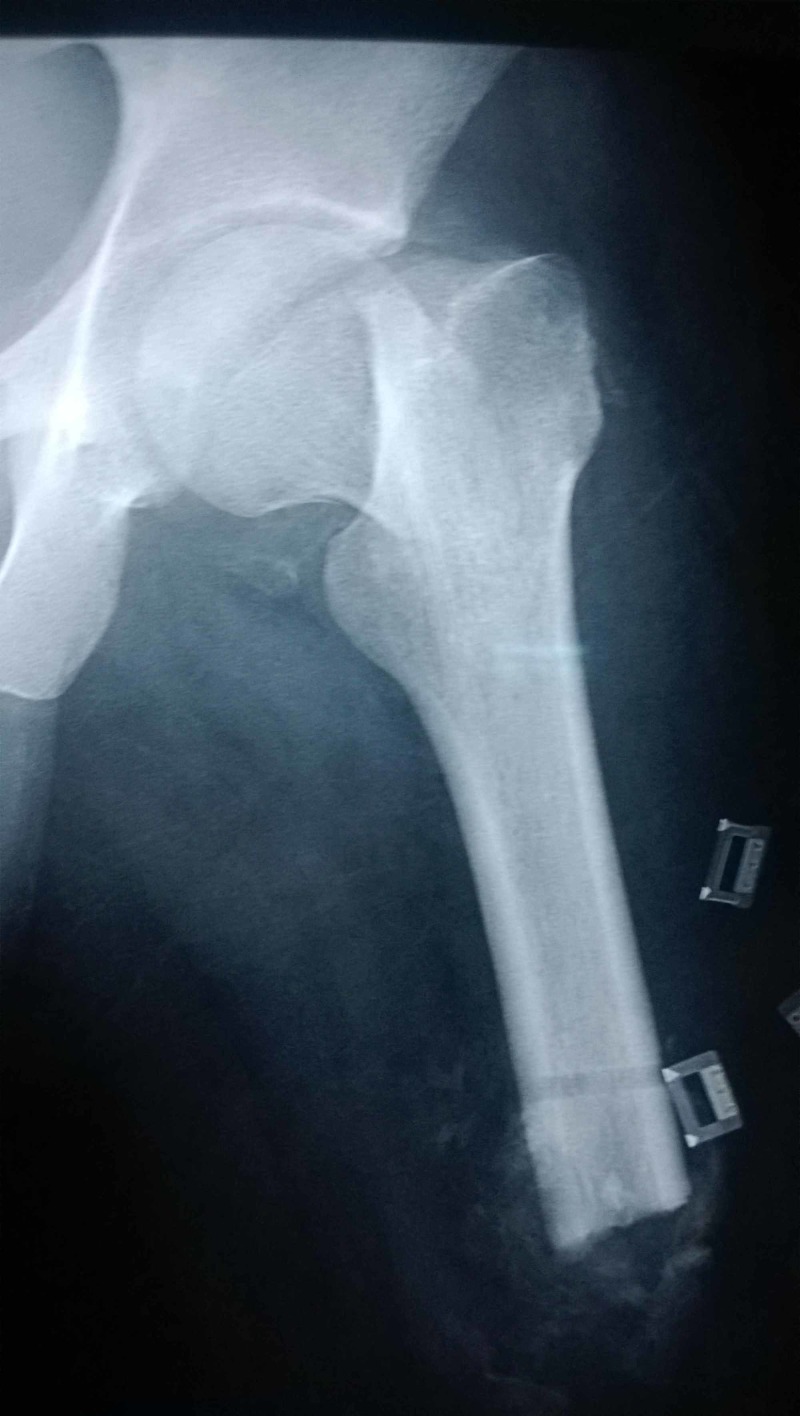
Antero posterior radiograph of patient B with a left sided femoral neck fracture. Note the overriding of the shaft fragment relative to the neck.

The first patient (patient A) was a 25-year-old male who had an open fracture of the right tibia with popliteal artery injury and had previously undergone an attempt of limb salvage with vascular repair and external fixation. Finally, when the limb couldn’t be salvaged, a trans-femoral amputation was performed four weeks from the initial injury. While a femoral neck fracture was evident on initial radiographs, it was not fixed by the primary operating surgeon. On evaluating the patient’s radiographs two and half months after the initial injury (Figure 1, 2), the femoral head had no evidence of sclerosis or sub-chondral cysts suggesting that it was probably viable and it was decided to address the fracture by fixing it. The second patient (patient B) was a 32-year-old male who was referred to us when hip instability was noticed at the time of evaluation for prosthetic leg fitting. The patient had undergone trans-femoral amputation for a crushed left leg three months back. The recent radiograph showed fracture of the femoral neck with much proximal over-riding of the short stump (Figure 3). Again, the head appeared to be viable. The stump was smaller in length than that in patient A. In this case too, an attempt of salvage of femoral head was taken.

Aiming to use three 6.5 mm partially threaded cancellous screws (PTCS) and a non-vascular fibula strut graft, a configuration of inverted triangular screw placement with fibula in the centre was agreed upon by the surgical team. To allow for adequate space for central fibular graft placement, the screws were planned to be placed adequately away from the center i.e. as near to the cortex of the neck as possible.

**Figure 4:**
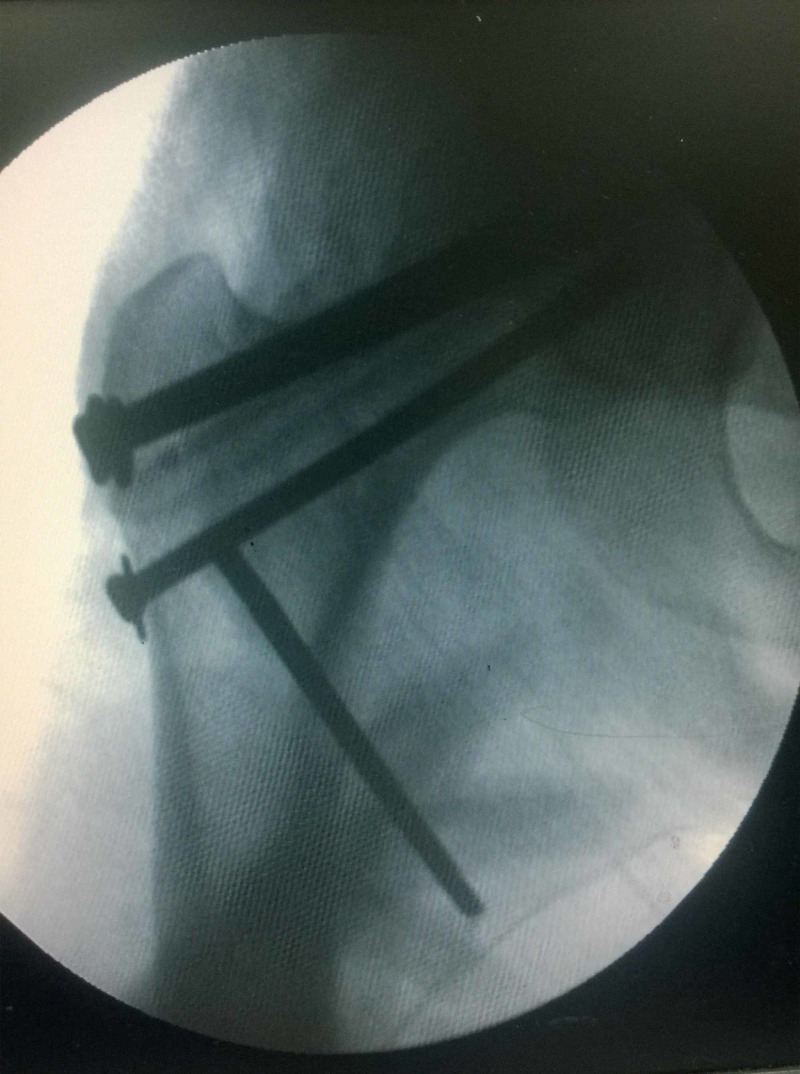
Intra-operative radiograph of patient A showing the three screws and the central fibula. The Schanz screw at the level of lesser trochanter was used to control rotation during reduction.

**Figure 5:**
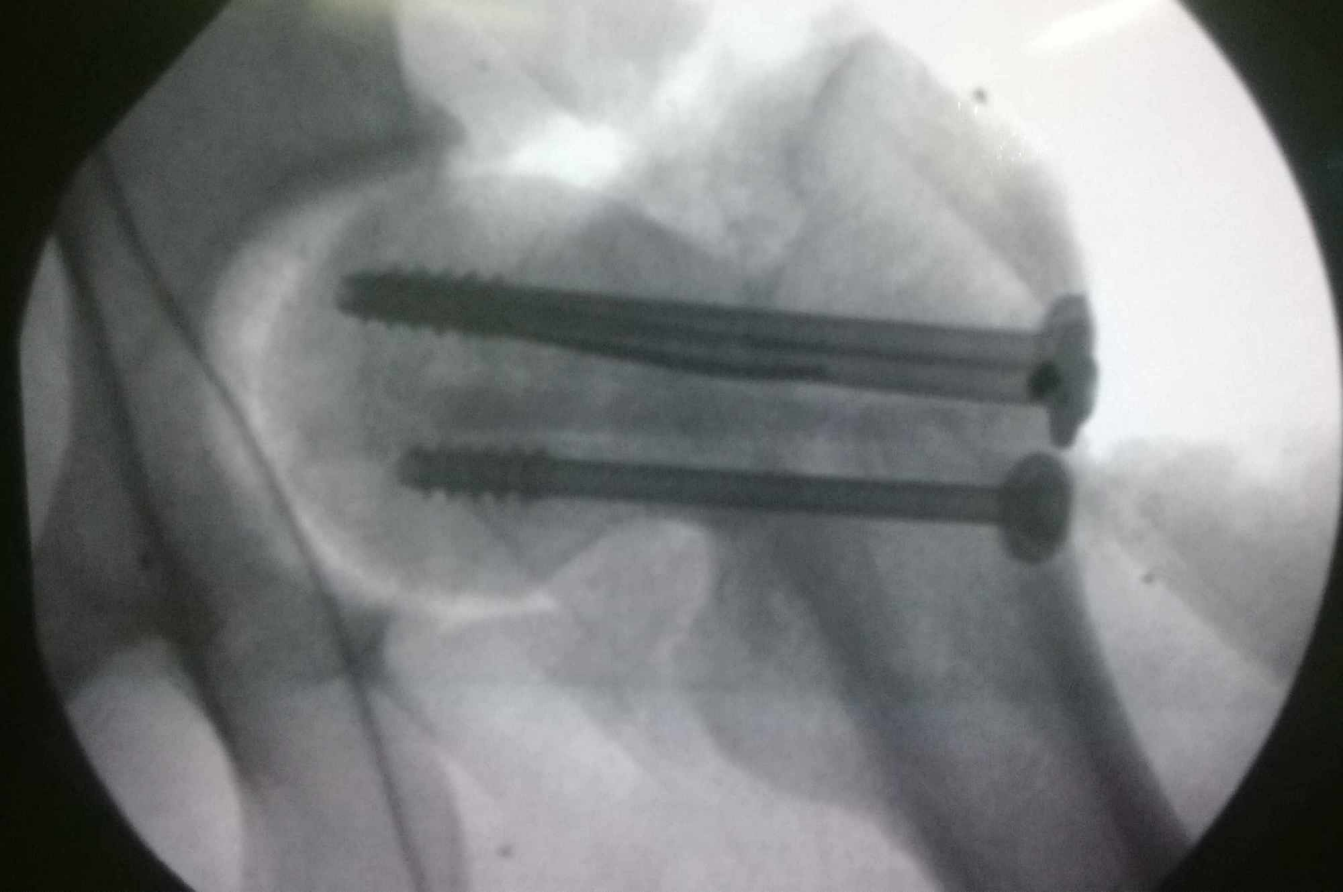
Intra-operative radiograph of patient B showing the screws and central fibula.

Both patients were operated under spinal anesthesia. The opposite leg was prepared for harvesting fibula auto-graft. The patient position was supine on a radiolucent table with a bump placed such that the ipsilateral buttock was lifted up allowing the gluteal muscles to hang. The Watson Jones approach was used. The fracture ends were noted to be sclerosed and smooth. There was fibrosis intervening between the fracture ends which was cleared. The fracture ends were freshened with a rongeur. A 4.5 mm Schanz screw was placed in the femoral shaft (as distally as permissible). A second 4.5mm Schanz screw was placed at the level of the lesser trochanter (LT) to control rotation. Two 2.5 mm smooth Kirschner wires (K-wires) were inserted into the femoral head. Through the Schanz screw placed in the shaft, linear traction was applied by a scrubbed assistant aiming to pull the fracture out to length. With linear traction along the shaft maintained, the fracture was reduced under vision by using the Schanz screw placed at LT and the K - wires placed in the head as “joy sticks”. In patient B, the distal fragment was lying high up and it did not descend adequately with traction. Capsular and soft tissue release was done to mobilize the distal fragment. Once the fracture was judged to be reasonably reduced, provisional fixation was done with two 3 mm K - wires. The adequacy of reduction was verified under image intensifier. Three guide wires were passed such that the wires were located at the periphery of the femoral neck. Three 6.5mm PTCS were then inserted. Another guide wire was then placed in the center of the neck (center of the triangle) and depth was measured. Measured length of fibula was harvested from the opposite leg. The sharp interosseous border of the fibula graft was nibbled to make it cylindrical. Using a dynamic hip screw (DHS) reamer, a channel was reamed in the neck. The fibula was then gently hammered into the reamed channel making sure that the graft did not break in the process (Figure 4 and 5 show the intra-operative images of patients A and B respectively). Wounds were closed over suction drains.

**Figure 6:**
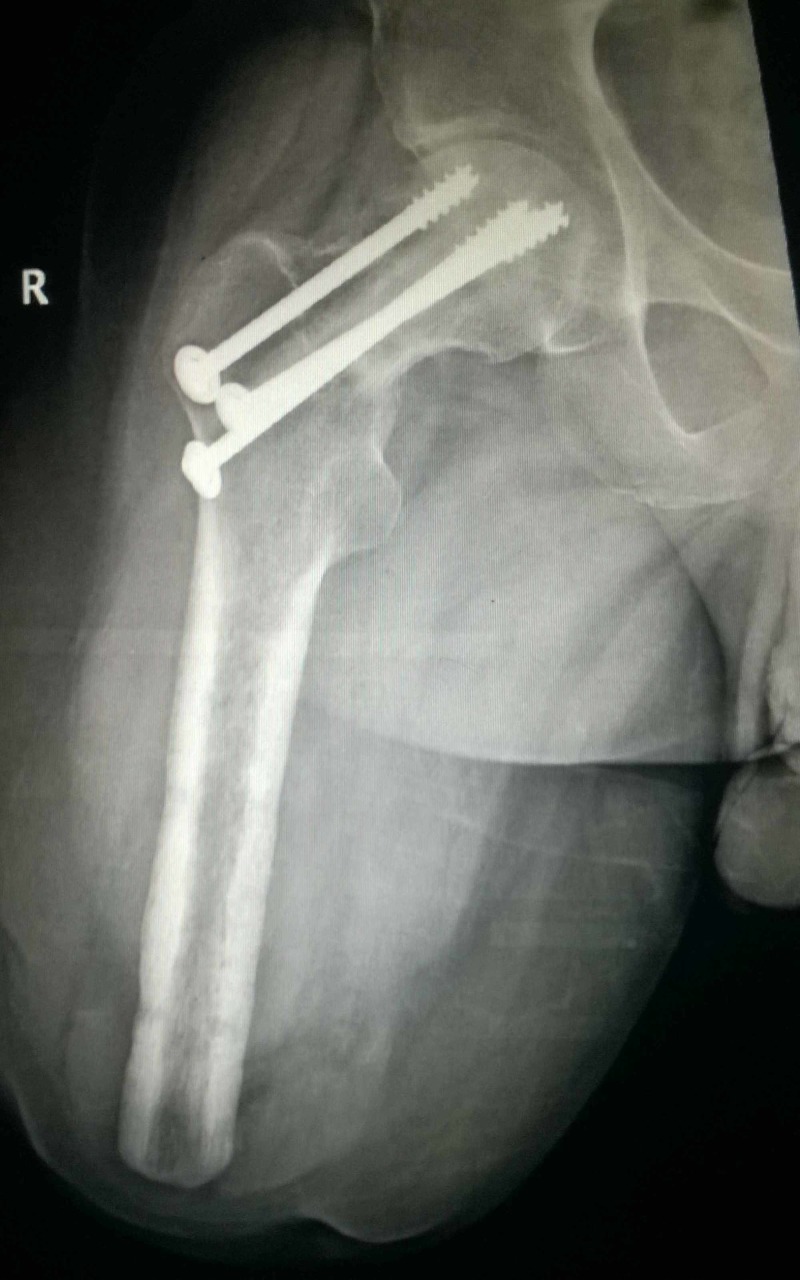
One and half years follow up antero-posterior radiograph of patient A showing the incorporation of fibula and healed fracture

**Figure 7:**
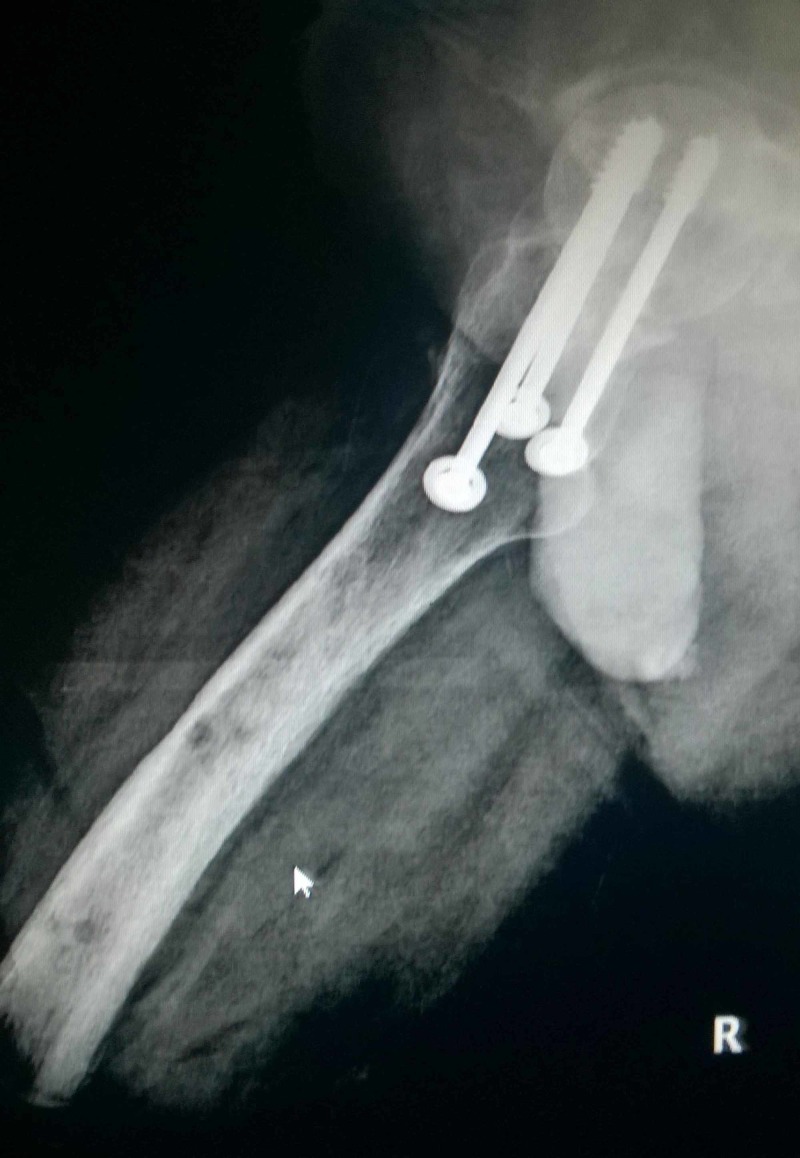
One and half years follow up lateral radiograph of patient A

**Figure 8:**
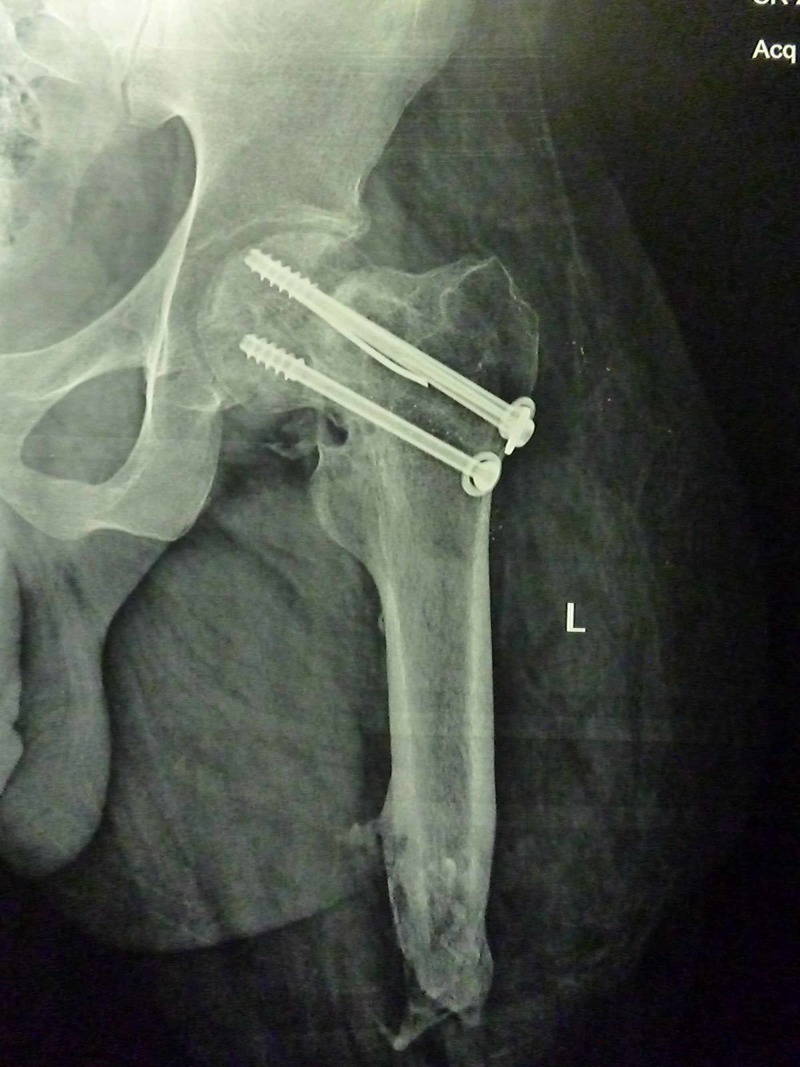
One year follow up antero-posterior radiograph of the patient A showing healed fracture and incorporated fibula. Also note the broken guide-wire which has not migrated.

**Figure 9:**
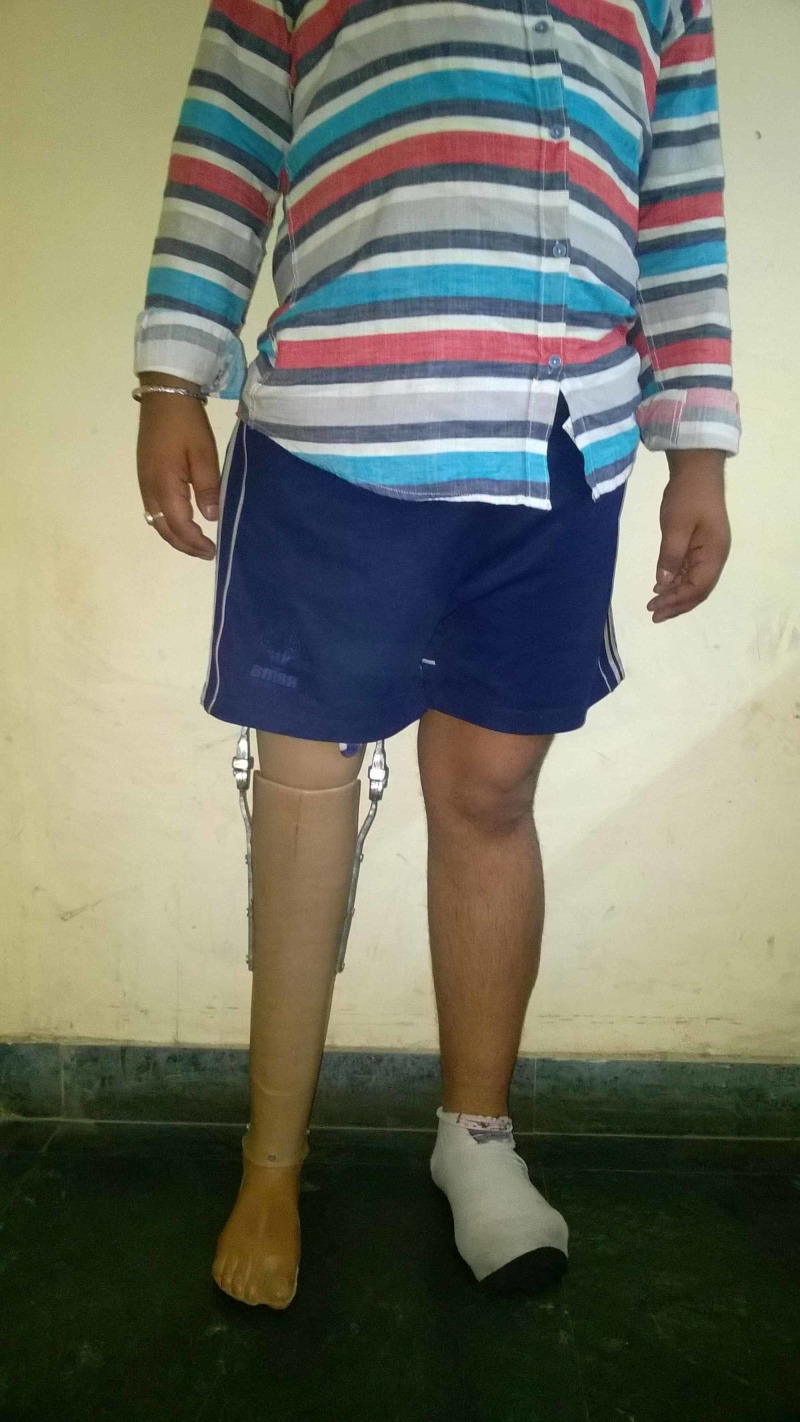
Clinical picture showing patient A standing comfortably on his prosthetic leg.

Patients were allowed ambulation with a pair of axillary crutches as soon as they became comfortable. Physiotherapy aiming at improving the hip abductors and extensors strength was started. Both the patients were referred to orthotist for prosthetic fitting. Follow up was done at one, three, six months and one year after surgery. At the latest follow up, one and half years for patient A and one year for patient B, both the patients were pain free. Radiographs obtained during their last follow-up showed union across the fracture site and good incorporation of the fibular graft (Figure 6 and 7 are of patient A while Figure 8 is of patient B). While the contour of the femoral head was completely maintained in patient A in his last follow-up, there was evidence of some collapse in patient B. At the last follow-up visit, patient A had adapted well to the use of prosthetic limb and was ambulating without any crutch support (Figure 9). Patient B is yet to adapt to his prosthesis.

## Discussion

Femoral neck fracture may be missed initially if it is associated with other injuries. Fractures associated with vascular injuries are not uncommon and if the initial evaluation is not meticulous, one may miss an associated fracture. In case a femoral neck fracture is missed, or management is delayed, the chances of it going into non-union or avascular necrosis (AVN) increases [8,9]. Neglected femoral neck fracture should be practically managed like a femoral neck non-union. Inter trochanteric valgus osteotomy and fixation with angle blade plate (ABP) or dynamic hip screw (DHS) aiming to convert shearing forces to compression forces is one of the methods described for management of femoral neck non unions [10]. Other described methods include the use of screws and fibula (vascularized or non-vascularized), muscle pedicle grafts and arthroplasty [11, 12]. An attempt to salvage the native femoral head should be undertaken in young adults. The muscle pedicle grafts and vascularized fibula grafting techniques have been reported as having good outcomes but are technically demanding. When using vascularized fibula, it is believed that the fibula provides mechanical strength and simultaneously stimulates fracture union while getting incorporated as a biological graft but using a vascularized fibula needs experience with microvascular surgical techniques [12]. Non-vascularized fibula is practically a dead bone but still acts as a strut to provide mechanical support. Good results have been reported in neglected femoral neck fractures managed with open reduction and internal fixation with compression screws and free fibular graft [13]. The screws provide immediate strength to the construct. It is postulated that the free fibula with osteogenic cells in its marrow acts to stimulate healing response.

Fractures in amputees are difficult to manage as the conventional means of management cannot be directly applied, and the surgeon must improvise in such cases [14]. The issues of positioning, surgical technique, implant choice and post-operative rehabilitation all must be addressed in these patients. In acute femoral neck fracture in a young transfemoral amputee, cancellous screw fixation has been reported by Freitas et al [3]. In older amputees, a hemi or total hip arthroplasty is preferable can be safely performed as has been reported previously [4-6].

Neglected femoral neck fracture in above knee amputees presents a particularly difficult scenario for management as the small size of femoral stump and contractures of surrounding muscles can make the reduction difficult. Meena et al have performed valgus osteotomy and used a DHS for neglected femoral neck fracture with good result [7].

In our patients, co-existing vascular injury probably led to missing of femoral neck fracture. As both the patients were young and the femoral heads had no signs of AVN on radiographs, the management was planned with an aim of salvaging the hips rather than replacing them. Neglected femoral neck fractures are frequently managed with two screws and a fibula graft at our institute. The overall short-term outcome can be reported to be good as the fractures united well with patients reporting no pain during their last visit. We believe that the technique of using ‘joysticks’ for reduction and fibular graft for augmentation of fixation is simple but reproducible for use in select circumstances especially if the stump is too small for ABP or DHS placement or if these implants are unavailable or the surgeon is comfortable in using fibula and screw combination.

## Conclusions

Careful evaluation of patients and their radiographs is a must in patients with lower limb injuries to avoid missing out on fractures, especially femoral neck fractures. Management of neglected femoral neck fracture in young above knee amputees using a fibular graft with three screws is a reasonably good method of salvaging the femoral head which can be performed comfortably by orthopedic surgeons dealing with femoral neck non unions.

## References

[1] Boulton CL, Pollak AN (2015). Special topic: Ipsilateral femoral neck and shaft fractures--does evidence give us the answer?. Injury.

[2] Cole PA, Shafiq B (2013). The diagnosis and management of musculoskeletal trauma. Manual of Orthopaedics.

[3] Freitas A, Souto DRM, da Silva JF, Dantas BR, de Paula AP (2015). Treatment of an acute fracture of the femoral neck in a young female adult with a transfemoral amputation: A case report. JBJS Case Connect.

[4] Ma C, Lv Q, Yi C, Ma J, Zhu L (2015). Ipsilateral total hip arthroplasty in patient with an above-knee amputee for femoral neck fracture: a case report. Int J Clin Exp Med.

[5] Kandel L, Hernandez M, Safran O, Schwartz I, Liebergall M, Mattan Y (2009). Bipolar hip hemiarthroplasty in a patient with an above knee amputation: a case report. J OrthopSurg Res.

[6] Diamond OJ, Mullan CJ, McAlinden MG, Brown JG (2013). Total hip arthroplasty following an ipsilateral above knee amputation. Hip Int.

[7] Meena U, Meena R, S B, Gaba S (2015). Management of neglected femoral neck fracture in above knee amputated limb: A case report. Chin J Traumatol.

[8] Soni A, Tzafetta K, Knight S, Giannoudis PV (2012). Gustilo IIIC fractures in the lower limb: Our 15-year experience. J Bone Joint Surg Br.

[9] Lu-Yao GL, Keller RB, Littenberg B, Wennberg JE (1994). Outcomes after displaced fractures of the femoral neck. A meta-analysis of one hundred and six published reports. J Bone Joint Surg Am.

[10] Khan AQ, Khan MS, Sherwani MK, Agrawal R (2009). Role of valgus osteotomy and fixation with dynamic hip screw and 120° double angle barrel plate in the management of neglected and ununited femoral neck fracture in young patients. J Orthop Traumatol.

[11] Elgafy H, Ebraheim NA, Bach HG Revision internal fixation and nonvascular fibular graft for femoral neck nonunion. J Trauma.2011.

[12] LeCroy CM, Rizzo M, Gunneson EE, Urbaniak JR Free vascularized fibular bone grafting in the management of femoral neck nonunion in patients younger than fifty years. J Orthop Trauma.2002.

[13] Jain AK, Mukunth R, Srivastava A Treatment of neglected femoral neck fracture. Indian J Orthop.2015.

[14] Bowker JH, Rills BM, Ledbetter CA, Hunter GA, Holliday P (1981). Fractures in lower limbs with prior amputation. A study of ninety cases. J Bone Joint Surg Am.

